# Sodium excretion and associated factors in urine samples of African descendants in Alcântara, Brazil: a population based study

**DOI:** 10.1080/0886022X.2017.1419967

**Published:** 2018-01-03

**Authors:** Elisângela Milhomem dos Santos, Dyego José de Araújo Brito, Isabela Leal Calado, Ana Karina Teixeira França, Joyce Santos Lages, Francisco das Chagas Monteiro Junior, Alcione Miranda dos Santos, Natalino Salgado Filho

**Affiliations:** a Department of Nursing, Federal University of Maranhão, São Luis, Maranhão, Brazil;; b Department of Nephrology, University Hospital of Federal University of Maranhão, São Luis, Maranhão, Brazil;; c Department of Health Sciences, Federal University of Maranhão, São Luis, Maranhão, Brazil;; d Department of Public Health, Federal University of Maranhão, São Luis, Maranhão, Brazil;; e Department of Medicine I, Federal University of Maranhão, São Luis, Maranhão, Brazil

**Keywords:** Sodium, excretion, hypertension, African Continental Ancestry Group, vulnerable groups

## Abstract

In most countries, salt intake has been excessive and constitutes one of the main risk factors for disease development, especially hypertension. Factors such as age, gender, sedentary lifestyle, smoking, African descent, obesity, dietary habits and family history of hypertension may be associated with high blood pressure. Studies show a positive association between the excretion of sodium and increased blood pressure. We evaluated the urinary excretion of sodium and associated factors in isolated urine samples of African descendants from remaining Quilombos. We performed a cross-sectional, population-based study with 1162 African descendants living in remaining quilombos in Alcântara, Maranhão, Brazil. Demographic, nutritional, clinical and laboratory data were analyzed. Urinary sodium excretion was estimated using the Kawasaki equation. A multivariate linear regression model was used to identify the variables related to sodium excretion. The average age was 37.6 ± 11.8 years and 51.2% were women. The prevalence of hypertension was 21.3%. The average urinary excretion of sodium was high, especially among the hypertensive (217.9 ± 90.1 vs. 199.2 ± 83.0 mmol/d; *p* = .002). After an adjusted analysis, only the waist circumference (odds ratios (OR) = 1.16; confidence intervals(CI)95%: 1.03–1.30), triglyceride (OR = 1.13; CI95%: 1.05–1.22), systolic blood pressure (OR = 1.19; CI95%: 1.08–1.32) and Chronic Kidney Disease Epidemiology (CKD-EPI;OR = 1.24; CI95%: 1.15–1.35) remained related to urinary sodium excretion. African descendants had a high rate of sodium excretion, especially among those who had hypertension. Abdominal adiposity, triglyceride and systolic blood pressure levels and renal function by CKD-EPI equation were associated to urinary sodium excretion.

## Introduction

High sodium intake is one of the main risk factors for the development of diseases such as strokes, left ventricular hypertrophy, chronic kidney disease and especially hypertension [1]. Association between sodium intake and hypertension has been showed since the 1950 s in different populations. These studies demonstrate positive linear correlation between the prevalence of hypertension and average intake of sodium [2,3].

The excessive consumption of salt has been observed worldwide, ranging from 9 to 12 g per person per day [4]. The WHO recommends a maximum 5 g of salt (2000 mg of sodium) per day for adults [5].

Sodium excretion in a 24 h urinary sample is considered the gold standard for evaluation of sodium intake. However, the difficulties associated with the accuracy of urine collection may interfere with the results, especially in population studies. Thus, the estimate of sodium intake from the collection of random urine, at a population level, has been increasingly used as a convenient and affordable alternative. Several formulas have been proposed to convert sodium in spot urine into a 24 h excretion estimation using creatinine to monitor urinary concentration [6].

Kawasaki et al. [7] and Tanaka et al. [8] proposed formulas based on random urine samples in Japanese population. Estimates of urinary sodium excretion using these formulas were close to the 24 h excretion estimation. The authors concluded that the collection of random urine is a suitable alternative to the 24 h urine collection in population surveys.

Brazilian studies that evaluate the consumption of salt are scarce, mainly in minority groups where there are differences in the amount of salt intake. These differences may be attributed partly to the evidence of changes in eating habits and the westernization. These factors may result in diseases and death by hypertension and other cardiovascular diseases in future [9]. Thus, we aimed to evaluate the sodium excretion and associated factors in random urine samples of African descent individuals who live in Quilombola communities in Alcântara, Maranhão, Brazil.

## Methods

This is a cross-sectional population-based study that included African descendants living in the city of Alcântara, Maranhão, Brazil. This study was part of the research project ‘Prevalence of chronic kidney disease in quilombo communities of Alcântara, Maranhão state(PREVRENAL)’. The PREVRENAL is a population-based cohort which evaluated, during July 2012 to April 2013, 1162 African descent individuals in 32 remaining Quilombo communities from Alcântara, Northern Maranhão. This study was approved by the research ethics committee at University Hospital, Federal University of Maranhão (HUUFMA), São Luís, Maranhão, Brazil.

Quilombos can be defined as ethnic and racial groups according to self-award criteria, with its own historical trajectory, endowed with specific territorial relations with presumption of descent from Afro-Brazilian slaves. In general, Quilombos have a certain degree of geographical isolation and cultural practices that have a strong relationship with the past [10].

The participants were selected by probabilistic sampling, per conglomerates in two phases. The first consisted of the identification of the census sectors (139 Quilombos); 32 of these sectors were then selected. The homes in each sector were chosen at random sampling, based on the calculation of the sample size. From the selected houses, all individuals aged 18 y or above were invited to participate in study. To those who accepted, explanations about the purpose of the study were given and they signed an informed consent (IC).

As a non-inclusion criteria, the following was adopted: individuals under 18 and over 59 y of age, pregnant women, patients with consumptive chronic diseases (cancer or acquired immunodeficiency syndrome), hematological diseases, autoimmune diseases, systemic infection or genitourinary tract infection, chronic and/or acute kidney disease under dialysis and those using immunosuppressive drugs or with thyroid disorders based on clinical history and physical examination.

In order to calculate the sample size, a pilot study with 76 African descendants from the studied population was conducted to estimate the average rate of sodium excretion. Assuming an average sodium excretion rate of 211.4 mmol/d, standard deviation of 71.4 mmol/d and a sampling error of 7 mmol/d, we obtained a sample size of 810 individuals. Considering eventual losses, the sample was increased by 10%. Nevertheless, owing to this study makes part of the PREVRENAL project, 1162 African descent people who met the inclusion criteria composed the sample.

The data collection occurred in two stages and was conducted by a multidisciplinary team. All directly involved researchers who were trained on implementation procedures, such as filling out forms, informed consent, measurement of blood pressure, anthropometric measurements, collection of laboratory tests and confidential information.

The initial stage of the study consisted of collecting household data through interviews using a structured questionnaire regarding demographic and socioeconomic data, lifestyle, previous medical history, medication consumption, use of health services and blood pressure measurements.

For the evaluation of the economic condition, the family monthly income was categorized into multiples of minimum wages (which in Brazil was approximately $US 296.19) and also the Criterion of Brazilian Economic Classification (CCEB), defining classes in metropolitan areas, were considered. The CCEB divides the population into seven classes: A1 and A2, B1 and B2, C, D and E, from best to worst [11]. The education of individuals was categorized as ≤8 y and >8 y of education completed.

After the questionnaire, the blood pressure (BP) was measured on the individual’s right arm while seated, resting for at least five minutes, using a digital sphygmomanometer (Omron® 705-IT, Japan), with an appropriate cuff covering approximately 80% of the arm area. Three measurements were made with a minimum interval of three minutes each. The first measurement was discarded and the average of the other two was obtained. To classify the BP, the proposal made by the VI Brazilian Guidelines on Hypertension was used [12].

After BP measurements, two collectors of urine, each previously identified by the name and numbers one and two, for the first and second urine of the collection day, respectively, were delivered to individuals. The first sample was used for urine sediment and the second for analysis of creatinine and sodium excretion. The subjects were instructed on collection procedures and storage. The need to fast for blood collection and clinical exams were also explained. They were also instructed to attend a location pre-determined by researchers the next day to deliver the urine samples and to collect blood.

In the second stage, one day after the interview, the collection of biological material and anthropometric measurements were conducted. Weight (Kg) was measured on a portable digital scale (Plena*^®^,* Brazil) and height (meters) with a stadiometer (Alturexata*^®^,* Brazil), both following the recommended techniques.

For anthropometric measurements, the body mass index (BMI) was calculated as the ratio of body weight (Kg) and the square of the height (m^2^). The classification recommended by the WHO was used. The following was considered: underweight, BMI <18.5 kg/m^2^; normal weight, BMI ≥18.5 and <25 kg/m^2^; overweight, BMI ≥25 and <30 kg/m^2^ and obese, BMI ≥30 kg/m^2^. The waist circumference (WC) in centimeters (cm) was measured at the midpoint between the last rib and the iliac crest at expiry, using a non-extensible anthropometric tape (Sanny^®^, Brazil). The adopted cutoff point was high risk, WC ≥94 cm for men and ≥80 cm for women and very high risk, WC ≥102 cm for men and ≥88 cm for women [13].

The blood and urine collected were identified. The blood was centrifuged and then placed in Styrofoam batteries with ice and transported to the reference laboratory where the analyses were performed.

Measurements of creatinine (reference value(RV): 0.40 to 1.40 mg/dL; Jaffé method); fasting plasma glucose (RV <100 mg/dL; automated UV hexokinase); triglycerides (RV: desirable <150 mg/dL, borderline 150 to 199 mg/dL, high ≥200 mg/dL; Enzyme/Trinder automated); total cholesterol (RV: desirable <200 mg/dL, borderline 200 to 239 mg/dL and high ≥240 mg/dL; Enzyme/Trinder automated); High-density lipoproteins-cholesterol (HDL-c ;RV: desirable ≥50 mg/dL for men and ≥40 mg/dL for women; Dextran/magnesium sulfate/sulphate automated); Low-density lipoproteins-cholesterol (LDL-c ;RV: desirable <130 mg/dL, borderline 130 to 159 mg/dL and high ≥160 mg/dL; Dextran/magnesium sulfate/sulfate automated).

The excretion estimation of the 24 h urinary creatinine (24 hPrCr) and the ratio Na/Cr in the spot voiding urine (SUNa/SUCr) were obtained for estimating the 24-h urinary Na (NaUr-24 h) and from the sodium/creatinine ratio in the casual urine (NaUr) were obtained for the estimation of sodium excretion of 24 h (Na24 h) in random urine sample by the Kawasaki Equations [7] that were validated in Brazil [14] as shown below:

- 24 h PrCr (mg) = [(15.12 × weight, kg) + (7.39 × height, cm) - (12.63 × age)] – 79.9 (Male);
- 24 h PrCr (mg) = [(8.58 × weight, kg) + (5.09 × height, cm) - (4.72 × age)] – 74.95 (Female);
- 24 hour urine Na excretion estimation (mg) = 23 × 16.3 × {[Na (mEq/L) in the spot urine/Cr (mg/dL) in the spot urine × 10] × PrUCr24h}^0.5^.

The renal function was evaluated by serum creatinine and estimated glomerular filtration rate (eGFR) by the equation proposed by the study Chronic Kidney Disease Epidemiology (CKD-EPI) described below [15]. For the diagnosis of the reduced kidney function, a eGFR of less than 60 mL/min/1.73 m^2^ was considered [16]. GF (mL/min/1.73 m^2^) = 141 × min (serum creatinine/κ, 1)α × max (serum creatinine/κ, 1) − 1.209 × 0.993 Age ×1.018 if women, 1.159 if blacks; with: κ = 0.7 for women and 0.9 for men; α = −0.329 for women and −0.411 for men; min is the minimum serum creatinine or 1 and max indicates the maximum serum creatinine or 1.

The collected data were analyzed using the STATA 14.0 software. The descriptive analysis was initially performed. Categorical variables were presented through frequencies and percentages, the numerical by means and standard deviation (mean ± SD). The normality of the numerical variables was assessed by the Shapiro-Wilk test.

Association between the categorical variables and the presence of arterial hypertension was analyzed using the chi-square test with a level of significance of 5%. To estimate the independent effect of variables on sodium excretion, a multivariate linear regression model was used and the variables tested in the model were those presenting *p* < .20 at non-adjusted analysis. Variables whose *p* values was less than.05 were considered statistically significant. Odds ratios (OR) and their confidence intervals were obtained (CI 95%). A logarithmic transformation was used in all variables to meet the normal distribution assumption required in linear regression models.

## Results

The study sample consisted of 1162 individuals with an African descent. According to Table 1, 51.3% were women with a mean age of 37.6 ± 11.8 y. 89.9% individuals declared themselves as black or brown and 58.6% had up to eight years of schooling. According to the CCEB, 88.7% were in classes D and E. It was observed that 84.8% had no fixed income and/or received up to the monthly minimum wage. The prevalence of alcoholism and smoking was 47.0 and 10.4%, respectively. Considering the anthropometric evaluation, it was observed that 46.4% of respondents were overweight according to the BMI and 45.6% had a risk of cardiovascular diseases according to WC.

**Table 1.** Demographic, socioeconomic and anthropometric characteristics of African descendants in Alcântara-MA, 2013.

	Total	Hypertensives	Normotensives	
Variable	*n* (%)	*n* (%)	*n* (%)	*p* value
Age (Years)				<.001
18–29	360.0 (31.0)	30.0 (12.2)	330.0 (36.1)	
30–39	279.0 (24.0)	46.0 (18.6)	233.0 (25.5)	
40–49	285.0 (24.5)	69.0 (27.9)	216.0 (23.6)	
50–59	238.0 (20.5)	102.0 (41.3)	136.0 (14.8)	
Gender				.521
Female	567.0 (48.8)	125.0 (56.0)	442.0 (48.3)	
Male	595.0 (51.2)	122.0 (44.0)	473.0 (51.7)	
Skin color				<.001
White	121.0 (10.0)	36.0 (14.6)	85.0 (9.3)	
Black or brown	1034.0 (89.5)	207.0 (83.8)	827.0 (90.5)	
Others	6.0 (0.5)	6.0 (1.6)	2.0 (0.2)	
Education				<.001
≤8 y	680.0 (58.6)	176.0 (71.3)	504.0 (55.2)	
>8 y	480.0 (41.4)	71.0 (28.7)	409.0 (44.8)	
CCEB				.560
B	2.0 (0.2)	1.0 (0.4)	1.0 (0.1)	
C	128.0 (11.1)	29.0 (11.8)	99.0 (10.9)	
D and E	1026 (88.7)	216.0 (80.8)	810.0 (89.0)	
Income (minimum wage)				.757
No fixed income	575.0 (49.5)	119.0 (48.2)	456.0 (49.8)	
Up to 1	410.0 (35.3)	85.0 (47.9)	325.0 (35.6)	
>1–2	130.0 (11.2)	32.0 (13.0)	98.0 (10.7)	
>2	47.0 (4.0)	11.0 (4.4)	36 (3.9)	
Smoker				.948
Yes	121.0(10.4)	26.0 (10.5)	95.0 (10.4)	
No/Former	1041.0 (89.6)	221.0 (89.5)	820.0 (89.6)	
Alcoholism				.310
Yes	546.0 (47.0)	109.0 (44.1)	437.0 (47.8)	
No/Former	616.0 (53.0)	1038.0 (55.9)	478.0 (52.2)	
BMI (kg/m^2^)				<.001
Slim	25.0 (2.1)	3.0 (1.2)	22.0 (2.4)	
Eutrophic	598.0 (51.5)	85.0 (34.4)	513.0 (56.1)	
Overweight	386.0 (33.2)	101.0 (40.9)	285.0 (31.1)	
Obese	153.0 (13.2)	58.0 (23.5)	95.0 (10.4)	
WC (cm)				<.001
Normal	633.0 (54.5)	92.0 (37.3)	541.0 (59.1)	
High risk	181.0 (15.6)	48.0 (19.4)	133.0 (14.5)	
Very high risk	348.0 (30.0)	107.0 (43.3)	241.0 (26.3)	

CCEB: Brazilian Economic Classification; BMI: body mass index; WC: waist circumference.

Considering the clinical characteristics of the study sample, 36.9% had an altered fasting glucose and serum levels above the total cholesterol desirable, i.e., 33.7%, LDL-C 29.5%, and triglycerides 22.8%. Those with HDL-C serum levels below the recommended were 43.9%. Considering the renal function through the CKD-EPI equation, 0.5% of subjects had a reduced glomerular filtration (Table 2).

**Table 2.** Characteristics according to biochemical variables of African descendants, Alcântara-MA, 2013.

	Total	Hypertensives	Normotensives	
Variable	*n* (%)	*n* (%)	*n* (%)	*p* value
Fasting plasma glucose (mg/dL)				<.001
<100 mg/dL	733.0 (63.1)	121.0 (49.0)	612.0 (67.0)	
≥100 mg/dL	428.0 (36.9)	126.0 (51.0)	302.0 (33.0)	<.001
Total cholesterol (mg/dL)				
<200	758.0 (66.3)	121.0 (49.8)	637.0 (70.7)	
≥200 and <240	276.0 (24.2)	74.0 (30.4)	202.0 (22.4)	
≥240	110.0 (9.6)	48.0 (19.8)	62.0 (6.9)	.705
HDL-c (mg/dL)				
≥40 for females and ≥50 for male.	651.0 (56.1)	136.0 (55.1)	515.0 (56.4)	
<40 for female and <50 for male.	509.0 (43.9)	110.0 (44.9)	398.0 (43.6)	
LDL-c (mg/dL)				<.001
<130	808.0 (70.6)	134.0 (55.1)	674.0 (74.7)	
≥130 and <160	224.0 (19.6)	63.0 (25.9)	161.0 (17.9)	
≥160	113.0 (9.9)	46.0 (19.0)	67.0 (7.4)	
Triglycerides (mg/dL)				<.001
<150	897.0 (77.2)	161.0 (65.2)	736.0 (80.4)	
≥150 and <200	141.0 (12.1)	36.0 (14.6)	105.0 (11.5)	
≥200	124.0 (10.7)	50.0 (20.2)	74.0 (8.1)	
Creatinine (mg/dL)				.298
0.40–1.40	1158.0 (99.7)	247.0 (100.0)	911.0 (99.6)	
>1.40	4.0 (0.3)	0.0 (0.0)	4.0 (0.4)	
CKD-EPI (mL/min/1.73 m^2^)				.468
≥60	1156.0 (99.5)	245.0 (99.2)	911.0 (99.6)	
<60	6.0 (0.5)	2.0 (0.8)	4.0 (0.4)	

HDLc: HDL-cholesterol; LDLc: LDL cholesterol; CKD-EPI: Chronic Kidney Disease Epidemiology.

The prevalence of hypertension was 21.3%. Hypertensive individuals, compared to normotensive, were elderly (41.3 vs. 14.9%; *p* < .001), less educated (71.3 vs. 55.2%; *p* = .001), were overweight (64.4 vs. 41.5%; *p* < .001) and had a high WC (43.3 vs. 26.3%; *p* < .001; Table 1). Furthermore, hypertensive patients had higher frequencies of change in biochemical parameters evaluated, except for HDL-C, creatinine and CKD-EPI equation showed no statistically significant difference (Table 2).

The average urinary sodium excretion of the sample was 203.1 ± 84.9 mmol/day. It was observed that hypertensive patients had a higher sodium excretion than people with a normal blood pressure (217.9 ± 90.1 vs.199.2 ± 83.0 mmol/day; *p* = .002).

In the unadjusted analysis, the following variables were related to the excretion of sodium: BMI (OR = 1.13; CI 95% (1.07–1.20)), WC (OR = 1.16; CI 95% (1.10–1.23)), fasting plasma glucose (OR = 1.08; CI 95% (1.02–1.15)), triglycerides (OR = 1.11; CI 95% (1.05-1.17)), SBP (OR = 1.18; CI 95% (1.11–1.25)), DBP (OR = 1.14; CI 95% (1.07–1.20)), CKD-EPI equation (OR = 1.09; CI 95% (1.03–1.15)) and hypertensive individuals (OR = 1.25; CI 95% (1.08–1.43)), Table 3).

**Table 3.** Unadjusted and adjusted regression of associated factors for the estimate of the urinary sodium excretion of African descendants, Alcântara-MA, 2013.

	Unadjusted			Adjusted		
Variable	OR	*p* value	CI 95%	OR	*p* value	CI 95%
Age	1.02	.519	0.96–1.08			
Women	0.97	.554	0.86–1.08			
BMI	1.13	<.001	1.07–1.20			
WC	1.16	<.001	1.10–1.23	1.16	.010	1.03–1.30
Fasting glucose	1.08	.005	1.02–1.15			
Triglycerides	1.11	<.001	1.05–1.17	1.13	.001	1.05–1.22
HDL-c	1.00	.930	0.94–1.06			
LDL-c	1.01	.840	0.95–1.07			
Average SBP	1.18	<.001	1.11–1.25	1.19	<.001	1.08–1.32
Average DBP	1.14	<.001	1.07–1.20			
CKD-EPI	1.09	.004	1.03–1.15	1.24	<.001	1.15–1.35
Hypertensive	1.25	.002	1.08–1.43			

OR: odds ratio; CI: confidence interval; BMI: body mass index; WC: waist circumference; HDL-c: high-density lipoproteins-cholesterol; LDL-c: low-density lipoproteins-cholesterol; SBP: systolic blood pressure; DBP: diastolic blood pressure; CKD-EPI: Chronic Kidney Disease Epidemiology.

After the adjusted analysis, only WC (OR = 1.16; CI 95% (1.03-1.30)), triglycerides (OR = 1.13; CI 95% (1.05–1.22)), SBP (OR = 1.19; CI 95% (1.08–1.32)) and CKD-EPI equation (OR = 1.24; CI 95% (1.15–1.35)), remained related to urinary sodium excretion (Table 3).

## Discussion

In this study, the estimated urinary sodium excretion of African descendants was considered high and was associated with waist circumference, triglycerides, systolic blood pressure and GFR estimated by CKD-EPI equation.

The average of 203.1 ± 84.9 mmol/day of sodium excretion observed among African. This value is similar to reported for the Brazilian population, which is approximately 4.5 g/day (195 mmol/day) [17], exceeding by more than two times the WHO recommendation, which is 2.0 g/day [4].

Vulnerability conditions of remaining quilombo communities were evidenced in this study by the concentration of people in the lower classes, lower income and poorer education levels, corroborating with Bezerra et al. [18]. Review articles show that socioeconomic differences play an important role in health and influence several factors such as access to health care, degree of information, knowledge, adherence to treatment [18,19] and the effect of salt on blood pressure. Molina et al. [20] in a study conducted in Vitória-ES, Brazil, with 2268 individuals, found that individuals belonging to lower classes had a greater additional salt intake than in higher classes. These authors inferred that sodium intake is strongly related to the socioeconomic level and may partially explain the higher prevalence of hypertension in lower socioeconomic classes.

It is worth highlighting that this African descendant population ensures its livelihood by planting manioc to produce flour, hunting, fishing and extracting native fruits such as babaçu, juçara and buriti. The region is located by the sea, creeks and rivers to catch fish and shellfish [10]. Although this type of food does not have high sodium content, its preparation contributed to a high sodium intake among evaluated African descendants. During the visits at their residence and at the dietary survey, it was noted that the consumption of dried shrimp (preserved in salt) with juçara and manioc flour is part of the eating habits of this population for main meals and even snacks.

High sodium excretion has also been observed in samples of Portuguese individuals with values of 202.3 ± 115.1 mmol/day [21]. Other studies found lower values. The Prevention of Renal and Vascular End-stage Disease study (PREVEND), with 7543 Dutch adults, had an average 24 h urinary sodium excretion of 142 ± 51 mmol, corresponding to a daily sodium intake of 3.3 g (8.2 g of sodium chloride) [22]. Asian individuals presented much lower values, an average of 125 ± 53.4 mmol/day [23].

In this study, estimation of urinary sodium excretion in spot urine and WC had a positive association (OR = 1.16), suggesting that high salt intake is an important factor in the occurrence of obesity. A longitudinal study conducted in a Danish population [Monitoring Trends in Cardiovascular Disease (MONICA)], after adjustment for confounding factors, showed a significant trend between salt intake and an increased energy consumption from all macronutrients. The increase of body fat was 0.24 kg and the WC of 0.18 cm per 100 mmol of 24 h urinary sodium excretion, during the six-year study period [24].

Our study found a positive association between hypertriglyceridemia and the estimation of urinary sodium excretion (OR = 1.13). According to the results from the KNHANES (Korea National Health and Nutrition Examination Survey,), from 2007 to 2013, the prevalence of high-TG in the population aged over 30 years had increased, reaching 17.1% in 2013 [25]. The relationship between sodium and triglyceride excretion was linear in Korean individuals and triglyceride levels were significantly higher in the highest quartile (*p* < .001) [26].

In this current study, Afro-descendants with GFR >60 mL/1.73 m^2^/min presented a 24% risk of excreting more sodium when compared to those with lower GFR (OR = 1.24).

African Americans are more prone to sodium retention and salt-sensitive hypertension than Caucasians [27]. According to Price et al. [28], even healthy African Americans have a 10% lower renal plasma flow than Caucasians at the same age, probably because of a more activated intrarenal renin-angiotensin system (RAS). Besides that, Afro-descendants present a lower nephron number when compared to Caucasians [29].

Impaired excretion of sodium is often present in patients with CKD, which leads to elevation of blood pressure and proteinuria, glomerular hyperfiltration as well as low response to rennin angiotensin aldosterone system (RAAS) blockade. In order to preserve renal function, lowering the intake of sodium to less than 2 g per day is recommended (corresponding to 5 g of sodium chloride) in adults, unless contraindicated, that is, patients with salt-losing nephropathies, postural hypotension and volume contraction who do not have heart failure [30].

Our findings also reveal a direct relation between systolic blood pressure and the estimate of urinary sodium excretion (OR = 1.19). The literature reports a positive association of salt intake, urinary sodium excretion and prevalence of hypertension [31].

Law et al. [32] showed a linear and direct correlation between blood pressure and salt intake, emphasizing that the effect of salt on blood pressure is intensified by age and by basal blood pressure. Similarly, Xu et al. [33] observed a positive linear correlation between salt intake and systolic blood pressure in Chinese from Yantai, in all adjusted and unadjusted models (*r* = 0.16, *p* = .01). Each 100 mmol/day in sodium intake was associated with a of 4.0 mmHg increase in systolic blood pressure.

The Prospective Urban Rural Epidemiology (PURE) Study involving 157,543 adults showed increases of 2.11 mmHg in the systolic blood pressure (SBP) and 0.78 mmHg in the diastolic blood pressure (DBP) for each 1 g increase in the estimate of sodium excreted. The tendency of this association was stronger with an increased sodium intake. An increase of 2.58 mmHg in SBP per excretion gram >5 g/d was observed [34].

According to Forman et al. [35], in a population-based cohort, they showed that a greater sodium intake is independently associated with higher increases in markers of endothelial dysfunction over time, suggesting that a continuous diet with high sodium content may lead to biological changes that favor the development of hypertension.

Otherwise, in a meta-analysis, it was shown that the reduction in dietary salt intake has no effect on blood pressure and that the greater the restriction, the greater the pressure drop [36]. In a randomized double-blind study conducted by He et al. [37], with individuals with isolated and combined systolic hypertension, the authors demonstrated that, with a low reduction in sodium intake, there was a drop of the systolic blood pressure and this was highly significant. The drop of the diastolic pressure was not statistically significant.

The estimate of sodium intake from the collection of random urine, at a population level, is increasingly used as a convenient and affordable alternative, although the 24 h urine collection is the ‘gold standard’. Nevertheless, there are limitations regarding the fact that random urine samples may indicate poor 24 h urinary excretion according to individual variations [6].

Thus, Ji et al. [38], suggest that more research is needed to measure the accuracy and adequacy of the random urine collection in different ethnic groups. Besides the operational difficulties for evaluating the salt intake, other aspects must be taken into account such as sodium sensibility and significant variation on daily intake. Thus, underestimation of the amount of ingested salt may be obtained because interpersonal differences, ethnicity and culture are not taken into consideration [19].

Many formulas have been proposed to convert sodium in random urine into 24 h excretion estimation by using creatinine to monitor urinary concentration. A formula, based on random urine of 591 Japanese individuals from an international multicenter epidemiological (INTERSALT) study, was proposed by Tanaka et al. [8]. A similar study with 159 Japanese people was performed but using a second morning urine [7].

In New Zealand, two formulas, one proposed by the Pan American Health Organization (PAHO) and the other derived from INTERSALT data, were tested on a sample with 101 healthy volunteers from a random and a 24 h urine sample. Estimates of urinary sodium excretion using these formulas were close to those of the 24 h excretion. The authors concluded that the collection of random urine is a suitable alternative to the 24 h urine collection in population surveys [6].

A study performed in different populations showed a higher intra-class correlation coefficient (ICC) between estimated and measured sodium excretion with Kawasaki equation compared to INTERSALT and Tanaka. The degree of bias for estimated and measured sodium was lower with Kawasaki (313 mg/day, 95% CI: 182–444) compared to INTERSALT (−872 mg/day, 95% CI: −728 to 1016) and Tanaka (−548 mg/day, 95% CI: −408 to −688) formulae (*p* < .001 and *p* = .02, respectively) [34]. A study for validation of Tanaka and Kawasaki formulae were performed in Brazil [14].

The strengths of this study include the large representative sample, the random selection of participants, which, in addition, are a vulnerable population. This was the first population-based study with African-descendant communities of Maranhão and Brazil that evaluated sodium excretion and associated factors.

The limitation of this study was the use of a cross-sectional design. Although the gold standard for measuring sodium excretion was not used, we utilized another equation validated in Brazil, where the population has a high degree of admixture as well as strong influence of Africans.

Whereas there is individual variation in sodium excretion during the day, the random sample tends to underestimate or overestimate its excretion. However, the estimate of urinary sodium can be a mean to monitor dietary sodium intake in population studies and low-income individuals, where the 24 h collection can be logistically difficult due to several factors [6].

## Conclusions

The sodium excretion estimation was high in random urine samples. Furthermore, WC, TG, SBP and estimated GFR by CKD-EPI equation were associated with urinary sodium excretion. These findings show that much greater attention should be given to the sodium intake because of health risks related to high intake of sodium.
